# Rapid Intraspecific Evolution of miRNA and siRNA Genes in the Mosquito *Aedes aegypti*

**DOI:** 10.1371/journal.pone.0044198

**Published:** 2012-09-21

**Authors:** Scott A. Bernhardt, Mark P. Simmons, Ken E. Olson, Barry J. Beaty, Carol D. Blair, William C. Black

**Affiliations:** 1 Department of Microbiology, Immunology and Pathology, Colorado State University, Fort Collins, Colorado, United States of America; 2 Department of Biology, Colorado State University, Fort Collins, Colorado, United States of America; National Institute for Communicable Diseases/NHLS, South Africa

## Abstract

RNA silencing, or RNA interference (RNAi) in metazoans mediates development, reduces viral infection and limits transposon mobility. RNA silencing involves 21–30 nucleotide RNAs classified into microRNA (miRNA), exogenous and endogenous small interfering RNAs (siRNA), and Piwi-interacting RNA (piRNA). Knock-out, silencing and mutagenesis of genes in the exogenous siRNA (exo-siRNA) regulatory network demonstrate the importance of this RNAi pathway in antiviral immunity in *Drosophila* and mosquitoes. In *Drosophila*, genes encoding components for processing exo-siRNAs are among the fastest evolving 3% of all genes, suggesting that infection with pathogenic RNA viruses may drive diversifying selection in their host. In contrast, paralogous miRNA pathway genes do not evolve more rapidly than the genome average. Silencing of exo-siRNA pathway genes in mosquitoes orally infected with arboviruses leads to increased viral replication, but little is known about the comparative patterns of molecular evolution among the exo-siRNA and miRNA pathways genes in mosquitoes. We generated nearly complete sequences of all exons of major miRNA and siRNA pathway genes *dicer-1* and *dicer-*2, *argonaute-1* and *argonaute-2*, and *r3d1* and *r2d2* in 104 *Aedes aegypti* mosquitoes collected from six distinct geographic populations and analyzed their genetic diversity. The ratio of replacement to silent amino acid substitutions was 1.4 fold higher in *dicer-2* than in *dicer-1*, 27.4 fold higher in *argonaute-2* than in *argonaute-1* and similar in *r2d2* and *r3d1*. Positive selection was supported in 32% of non-synonymous sites in *dicer-1*, in 47% of sites in *dicer-2*, in 30% of sites in *argonaute-1*, in all sites in *argonaute-2*, in 22% of sites in *r3d1* and in 55% of sites in *r2d2*. Unlike *Drosophila*, in *Ae. aegypti*, both exo-siRNA and miRNA pathway genes appear to be undergoing rapid, positive, diversifying selection. Furthermore, refractoriness of mosquitoes to infection with dengue virus was significantly positively correlated for nucleotide diversity indices in *dicer-2*.

## Introduction

RNA silencing, or RNA interference (RNAi), in plants and animals mediates normal growth and development [1], [2], controls or eliminates viral infection [3] and limits transposon mobility in both germ line [4] and somatic cells [5], [6]. RNA silencing involves small RNAs that are 21–30 nucleotides (nt) in length and are divided into three main classes: microRNAs (miRNAs), exogenous and endogenous small interfering RNAs (exo- and endo-siRNAs), and Piwi-interacting RNAs (piRNAs).

Much of what we know about RNAi in insects has been elucidated in *Drosophila melanogaster*, where the biogenesis and regulatory functions of each of the small RNA classes have been separated into distinct pathways [7]. The exo-siRNA pathway has a central role in *Drosophila* antiviral immunity [8],[9] and is initiated by Dicer-2 (Dcr2). Dcr2 is an RNase III family protein that recognizes cytoplasmic long dsRNA and cleaves it into ∼21 bp siRNAs [10], [11]. The siRNAs, in association with Dcr2 and the dsRNA-binding protein R2D2, are loaded into a multi-protein RNA-induced silencing complex (RISC), which contains Argonaute-2 (Ago2) [12], [13], [14]. In the effector stage of the pathway, the RISC unwinds and degrades one of the siRNA strands and retains the other strand as a guide for recognition and sequence-specific cleavage of viral mRNA, mediated by the “slicer” endonuclease activity of Ago2 [15], [16], [17], [18].

MicroRNAs (miRNAs) are 22–23 nt RNA guides that regulate cellular functions such as differentiation and development and metabolic homeostasis. Although only invertebrates have siRNAs, both vertebrates and invertebrates have miRNAs, which are transcribed from the cellular genome as primary miRNAs by RNA polymerase II and are processed sequentially by two distinct endonucleases in the RNase III family, nuclear Drosha and cytoplasmic Dicer 1 (Dcr1), the only ortholog of the *dcr* gene family in mammals. Dcr1 processes pre-miRNA to imperfectly base-paired duplex miRNA with ∼23 nt strands and acts with the dsRNA-binding protein R3D1 to load the miRNA guide strand into an Argonaute-1 (Ago1)-containing RISC [13], [19]. Typically miRNAs recognize targets in the 3′ non-coding region of cellular mRNAs by imperfect complementarity and inhibit their translation [20].

There is currently a great deal of interest in identifying genes that condition the ability of arthropods to transmit RNA viruses that are pathogenic to humans and domestic animals. Of particular interest is the mosquito *Aedes aegypti*, which is an important vector of a number of pathogenic arthropod borne viruses (arboviruses), including the dengue viruses (DENV1-4), yellow fever virus and chikungunya virus, and is also a tractable genetic system with which to identify candidate genes [21]. *Aedes aegypti* is distributed in all subtropical and tropical regions of the world. Most importantly, *Ae. aegypti* populations demonstrate a great deal of variation in their susceptibility to arboviral infection [22].

Several lines of evidence suggest the importance of the exo-siRNA pathway in antiviral immunity in *Drosophila* and mosquitoes. *Drosophila* with mutations in or depletion of known exo-siRNA pathway components are hypersensitive to RNA virus infections and develop a dramatically increased viral load [9], [23], [24]. Increases in arboviral replication occur after knock-down of one or more genes in the exo-siRNA pathway [25], [26]. siRNAs derived from the infecting virus genome (viRNAs) have been discovered and characterized in infected insects [27], [28], [29], [30]. Many insect pathogenic viruses encode suppressors of RNAi that counteract insect immunity [31].

Noting that interaction between RNA viruses that encode suppressors of RNAi and their insect hosts may lead to a co-evolutionary “arms race” and directional selection on RNAi genes, Obbard et al. [32] undertook a comparative study of the rates of amino acid evolution in exo-siRNA and miRNA pathway components in three species of *Drosophila*. They showed that among *Drosophila* species, the ratio of replacement to silent amino acid substitutions (*w* = K_A_/K_S_) among the exo-siRNA genes *dcr2*, *r2d2*, and *ago2* is much greater than *w* among their miRNA-pathway counterparts *dcr1*, *r3d1*, and *ago1*. In fact it was shown that Dcr2, R2D2, and Ago2 are among the fastest evolving 3% of all *Drosophila* proteins [32]. Recent selective sweeps in *ago2* have reduced genetic variation across a region of more than 50 kb in the genomes of *Drosophila melanogaster*, *D. simulans*, and *D. yakuba*, and it was estimated that selection has fixed adaptive substitutions in this gene every 30–100 thousand years [33]. The rapid evolution of exo-siRNA pathway genes compared with miRNA pathway genes supported a hypothesis for directional selection on host antiviral RNAi genes driven by evolution of virus-encoded suppressors of RNAi (VSRs) that enable viruses to evade RNA silencing [32], [34].

More than 50 VSRs encoded by plant and insect pathogenic viruses have been described. The Flock House virus (*Nodaviridae*) protein B2 is one of the best characterized animal VSRs [35]. B2 binds both siRNA duplexes and long dsRNA, thereby preventing dsRNA binding by proteins in the exo-siRNA pathway. No arbovirus VSRs have been identified [31], [36], [37]; however, mosquito-borne alphaviruses were engineered to express B2 and then used to infect mosquitoes orally or by injection [28] [38]. These mosquitoes had reduced pools of viRNAs, increased infectious virus titers and, most importantly, greatly decreased survival rates relative to mosquitoes infected with non-recombinant viruses.

The ability of arboviruses to cause persistent, non-cytopathic infections in both mosquito cells and mosquitoes despite the RNAi response has led to speculation about arboviral mechanisms of immune suppression or evasion in insect cells. Entomopathogenic VSRs are generally virulence factors that increase the likelihood of virus transmission by killing the insect hosts; however, pathogenic rather than persistent infections of mosquitoes by arboviruses would be detrimental to transmission and maintenance in nature. Thus, arboviral mechanisms of evasion are unlikely to involve VSRs that increase virulence, but could involve strategies such as rapid evolution of genome ‘decoy’ regions [39] or RNAi-escape mutations [29].

A recent review concluded that mosquito RNAi is the major innate immune pathway controlling arbovirus infection and transmission in mosquitoes in a similar way to *Drosophila* antiviral immunity [40]. However, there has been no characterization and comparison of the molecular evolution of miRNA and exo-siRNA genes in a mosquito. Herein we describe the intraspecific patterns of molecular evolution of *ago1*, *ago2*, *dcr1*, *dcr2*, *r3d1*, and *r2d2* within and among collections of *Ae. aegypti* from throughout its geographic range. We tested whether the interspecific evolutionary patterns of small RNA pathway genes among *Drosophila* species [32] are also apparent intraspecifically in *Ae. aegypti*. We compared nearly complete sequences of all exons in each of the six genes from 104 individual *Ae. aegypti* collected in six geographically distinct sites throughout the mosquito's range. We determined if amino acids encoded by the six major genes in the two pathways appear to be under positive selection by performing a fixed-site phylogenetic analysis by maximum likelihood (PAML) [41] on each of the six genes. Identified variable sites were further characterized as to the likelihood that they would alter protein structure or function using the program SIFT (Sorting Intolerant From Tolerant) [42]. Finally, we sought to determine if sequence diversity in the six genes is correlated with susceptibility to infection with DENV2 by calculating Pearson's correlation coefficients between vector competence data gathered in previous studies for four of the six mosquito collections [43], [44], [45] and various measures of genetic diversity.

## Materials and Methods

### Mosquito strains and DNA extraction

**Figure 1:**
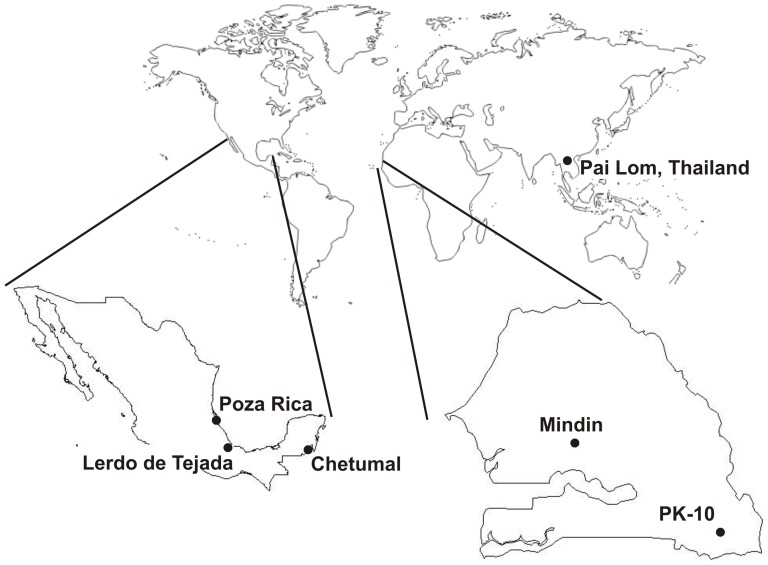
*Aedes aegypti* collection sites.

Six geographically distinct *Ae. aegypti* populations were analyzed in this study (Figure 1). DNA was analyzed from 18 *Ae. aegypti* individuals collected from Poza Rica, 18 from Lerdo de Tejada, and 18 from Chetumal in Mexico. Details on these collection sites and determination of their vector competence for DENV2 were published previously [43], [44]. DNA was analyzed from 20 mosquitoes from PK-10 (near Kedouguo) and ten from Mindin, Senegal [45]. All mosquitoes from PK-10 and five from Mindin were the *formosus* subspecies of *Ae. aegypti* as determined by the absence of silver scales on the first abdominal tergite [46]. Vector competence in *Ae. aegypti formosus* for flaviviruses tends to be lower than in subspecies *Ae. aegypti aegypti* [47], [48], [49]. The 20 *Ae. aegypti* from Pai Lom, Thailand were from a laboratory colony provided by Dr. L. C. Harrington at Cornell University. DNA was extracted from all 104 mosquitoes using the Qiagen DNeasy Blood and Tissue Kit (Valencia, CA).

### PCR amplification

**Figure 2:**
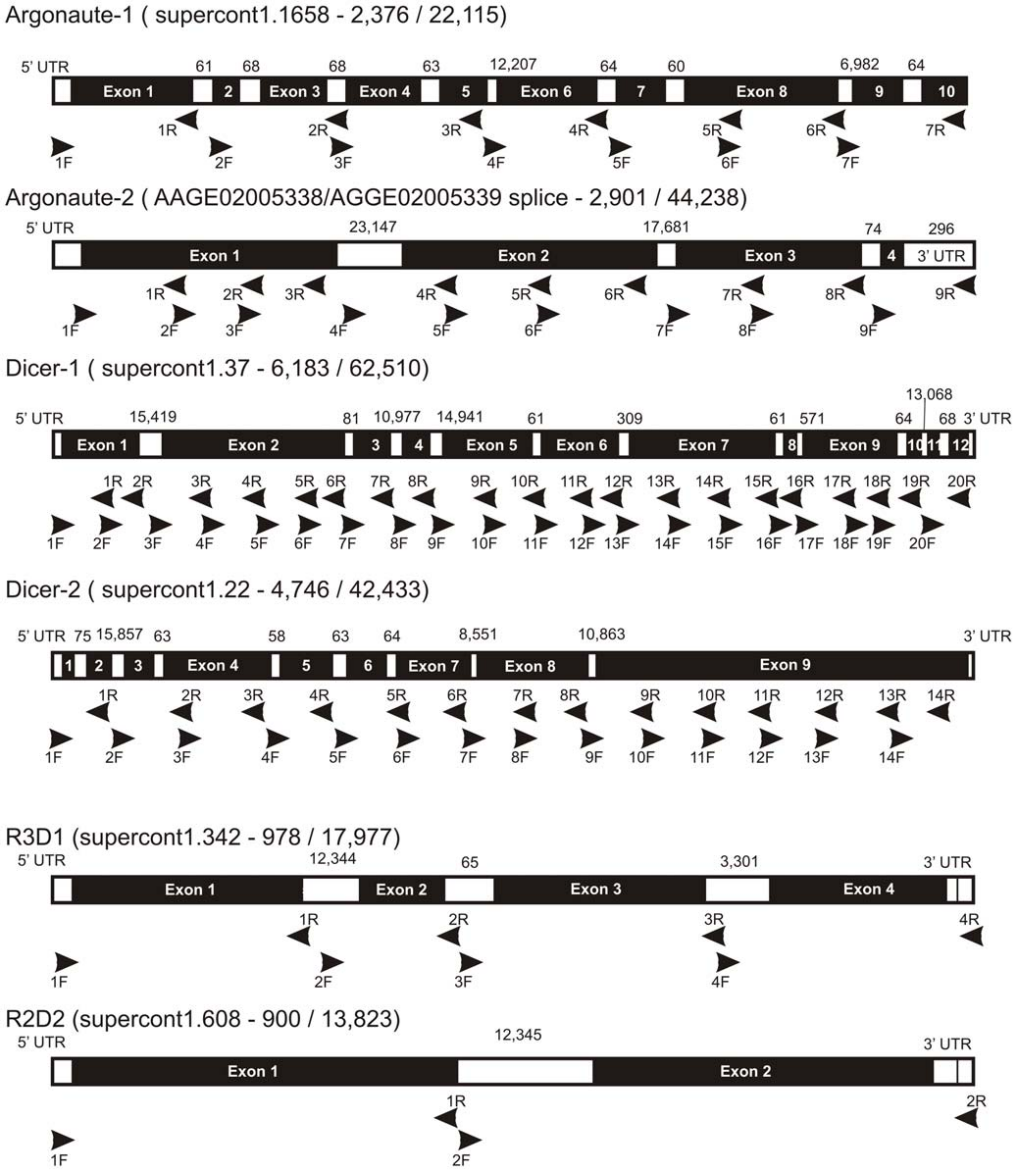
PCR primer locations on miRNA and siRNA pathway genes. Positions are numbered with respect to supercontigs in the *Ae. aegypti* genome project in VectorBase. Start position of each primer and lengths of amplicons are given in Table 1. Lengths of exons are given above each gene.

Primers for PCR (Table 1) were designed to amplify most of the exon regions of *dcr1*, *dcr2*, *ago1*, *ago2*, *r2d2* and *r3d1* genes. Primer locations and regions amplified with respect to supercontigs in the *Ae. aegypti* genome project in VectorBase (http://www.vectorbase.org/) are listed in Table 1 and shown in Figure 2. There were 56 amplicons analyzed in each of the 104 mosquitoes: *dcr1* (20 amplicons), *dcr2* (14), *ago1* (7), *ago2* (9), *r3d1* (4), and *r2d2* (2). In *dcr1*, 94% (6,183/6,581) of nucleotides were sequenced while in *dcr2*, 95% (4,746/4,976) of nucleotides were sequenced. In *ago1* and *ago2*, 96% (2,376/2,477) and 97% (2,901/2,979) of nucleotides were sequenced, respectively. In *r3d1*, 99% (978/989) of nucleotides and 94% (900/956) of *r2d2* nucleotides were sequenced.

**Table 1:** Primers for PCR amplification of most of the exon regions of *dcr1*, *dcr2*, *ago1*, *ago2*, *r2d2* and *r3d1*.

Gene	Amplicon ID	Forward/Reverse Primers	Start location	PCR product size	T_a_
**Ago1**	Ago1#1	TCGTGCTGCGTGCCAATCACTTCCA*	32	455	55.8
		AATRACTTAYCCATCGGGCGAACTG*			
	Ago1#2	CCTGGGCGGAGGTCGTG	487	440	55.8
		AGGAAATAGAATCAAGAAGGGGAGT*			
	Ago1#3	GCTTCCCTCTGCAGCTAGAAA	928	455	53.0
		GCGTCCTCCGTACTGTAACTTC			
	Ago1#4	GTGGCACGTTAAAATCAGC*	13,585	402	53.0
		TACCAGGAACACCCCAAT*			
	Ago1#5	TTCAACAGCGGAAGTCAA	14,007	428	53.0
		GCAACTCCCGAACCATAC			
	Ago1#6	GCAGCAGCACCGCCAGG	14,379	403	55.8
		CTTTCCCCGCATCAAACTCA*			
	Ago1#7A	CATTTGCTTCTAATTTCAG*	21,724	203	48.2
		TTATTTGTCCTTACCTGG*			
	Ago1#7B	CGCCCATTTGGTGGCATTC	21,886	236	50.4
		CTTTCTTTAAGCGAAGTACA*			
**Ago2**	Ago2#1	ACTGTATGATACTAAACGC*	5	428	50.4
		CGACGGTGACGACGAAT			
	Ago2#2	GCATTCGTCGTCACCGTCGCA	405	337	53.0
		AGCCGCATAGGCATTTTT			
	Ago2#3	TAGCGAGTTCACCAAGC	689	345	48.2
		AACGACTAAGGTTATCAT*			
	Ago2#4	TTCATATTTCTCTCTATTGC*	23,994	427	48.2
		AGGATACCGAAGTTGTTTGT			
	Ago2#5	CAACTTCGGTATCCTTCT	24,406	399	50.4
		TTCCCGTCTTGTAATCTCC			
	Ago2#6	GTACGGAGATTACAAGACGGGAAT	24,782	412	58.5
		CCAACAAAACTTACCTTGAGCCA*			
	Ago2#7	GCATTTTACAGATCAACGCCA*	42,911	368	48.2
		ACGAAGTTCTATGGTCAGTA			
	Ago2#8	CTGACCATAGAACTTCGTGC	43,261	428	53.0
		GCTACTCACTCTTTGATGTAGACGC*			
	Ago2#9	CGTCCTCTGAACATGAACAACCT	43,756	389	48.2
		AGTATCCTAGAGCCAACAA*			
**Dcr1**	Dcr1#1	GTCCTACGACGAGAATGGCTTAC*	14	430	53.0
		ATCACCAGCAGATTTACTT			
	Dcr1#2	TTGTGGCTATTTGGATCT	375	292	48.2
		AACCTGTTAGTGGACCTTA*			
	Dcr1#3	ATTTGCTACCAGCCCTAA*	16,012	444	48.2
		CATTGGAATACTGTTGGAAA			
	Dcr1#4	TTTTCCAACAGTATTCCAAT	16,435	453	48.2
		CATTCTGGTTGTAATAGTTTG			
	Dcr1#5	CAACCAGAATGATCCAGATGCTTTA	16,877	448	53.0
		CATGCGTTCGCTCAGATCCTCAC			
	Dcr1#6	CTTCCACCGCATATCATATTCTC	17,236	315	48.2
		TCTCCATATAGATAGC			
	Dcr1#7	TCGTTAGCGTAATTTGATAACAC*	17,603	347	55.8
		TCTTACTTACCACAATGTCTTCCT*			
	Dcr1#8	GGCTTGCCCATGCCTACGGAGAT	28,917	264	48.2
		GATGTTACAAGAATAGGTACT*			
	Dcr1#9	AAACTCATTTCTTTCCCTCAGATC*	44,077	442	55.8
		TTCCAATCAACGGTAACAACACT			
	Dcr1#10	ACGAGAAAGTGTTGTTACCG	44,490	448	48.2
		CTTGTAGGAAGAGCC*			
	Dcr1#11	GTGAATCGGAAAGGGGTGGCTCT	44,906	428	55.8
		ATCGCAGCAGATTTGTCA			
	Dcr1#12	ATCTGCTGCGATTGAGGAA	45,322	259	48.2
		TAGTGTAAACTTGTCATGTATAAAT*			
	Dcr1#13	GCCAGTGTTTCTCAACCTGTATT*	45,814	450	55.8
		GTCCCACAACTCCTAATGCGTT			
	Dcr1#14	AACGCATTAGGAGTTGTGGGACG	46,242	450	53.0
		GGTTGTCGGTCAGATTGTTGAGA			
	Dcr1#15	TCTCAACAATCTGACCGACAACC	46,669	410	50.4
		CGGAGTTCACTTACCTT*			
	Dcr1#16	GTATGCTTGTAGTTTGCTAGTCC*	47,080	199	50.4
		TTCACTTTTAACCATGTAGA*			
	Dcr1#17	GACAATGAAGAGGGCGAAAC	47,815	425	55.8
		AAGGCACTGTAACCATCCAAGA			
	Dcr1#18	TCTTGGATGGTTACAGTGCCTTC	48,218	297	53.0
		GGTGCCTGAAATACTTGTGG			
	Dcr1#19	GATTTCCACAAGTATTTCAG	48,490	306	53.0
		CTTACCTTCCACACAGCGTCCA*			
	Dcr1#20	ACCGAAGTATGATGGGACC	61,863	348	50.4
		GTGATATTTCAACGACGTTTGTT*			
**Dcr2**	Dcr2#1	CCGCTTGACAAATTTTTCAG*	53	312	48.2
		GCCCATACTCACTTATCCAG*			
	Dcr2#2	CCATTAACTGAAGGTG	16,101	441	48.2
		ATAGAGTAACATTCCAGAAAGACCG			
	Dcr2#3	GTGTAATCGGTCTTTCTG	16,510	394	53.0
		ACGCCAGTCTTAGCATTG			
	Dcr2#4	GCAGTTTGCGAAAGCCTG*	17,047	342	48.2
		AATGAAAGACATCACCCTTCTATCC*			
	Dcr2#5	TAAAAAGAATGAAACAAACG	17,451	405	48.2
		CCTTGGCGGTGAAAAACGGCG			
	Dcr2#6	ATTCCGCCGTTTTTCA	17,831	365	50.4
		AAATCCTTCCAATGACG			
	Dcr2#7	TCAGACCTCGGCAAACTA*	26,751	398	50.4
		AGAAATTCCTTCCACAGT			
	Dcr2#8	CCGAAATAGAACTTGCTC	27,070	373	48.2
		CGTAACATAACTTACCCGTAC*			
	Dcr2#9	CATTTGCTTTTCTTTTCAGTTCCTA*	38,272	394	53.0
		TGCTTTCCTTTCCGCTCCTTG			
	Dcr2#10	GAGCGGAAAGGAAAGCAG	38,649	394	50.4
		CTACGGGTACATCCAAGA			
	Dcr2#11	CAATCTTGGATGTACCCGTAG	39,022	357	55.8
		GAGGTGGTTGCCAGTCGT			
	Dcr2#12	CAACCACCTCTAGCAACG	39,369	411	48.2
		ATCTACTTCACGAATATCAA			
	Dcr2#13	CGCACAATGTCCTGAAGC	39,700	456	53.0
		GATCGGTAATTTCGTGTT			
	Dcr2#14	TTACCGATCAGGTGAAC	40,147	435	50.4
		AAACGAAACATTACTTAGCAC*			
**R3D1**	R3D1#1	AAAATCATTTCAGATGAGTA*	14	343	50.4
		GACTTACCATCCTTGTCCTG*			
	R3D1#2	GAAATATGTCATGTTTAAGG*	12,625	182	48.2
		CGCAGATGGAATAACTTACT*			
	R3D1#3	GTTCAATATGCTCACTATCA*	12,815	357	48.2
		TAAATCCCTACCACC*			
	R3D1#4	AGCGGGAAAACTATTACCAA*	16,389	360	50.4
		CCAGACAAGTACATAGACAT*			
**R2D2**	R2D25′	CTTGTGGTGTAGAATAATGG	16	603	48.2
		TAATCATCTCGTTGC			
	R2D2	ATACTTTAGATACCTCCCATTGACA*	13,713	659	50.4

Primer locations are numbered with respect to supercontigs in the *Ae. aegypti* genome project in Vector Base (Figure 2). An * indicates that all or part of that primer is located in an intron.

Single strand conformational polymorphism (SSCP) analysis was performed on all PCR products [50]. Gels were scored based on the SSCP banding patterns observed for each of the 56 amplicons for each individual mosquito in the study. Unique haplotypes were identified and recorded from each SSCP gel. Each unique haplotype for each of the 56 amplicons was sequenced on both strands on the ABI 3130×L Genetic Analyzer at the Proteomics and Metabolomics facility at Colorado State University. Sequences were obtained from at least two mosquitoes to test the sensitivity of the SSCP technique when a haplotype pattern occurred more than once.

### Sequence analysis

Forward and reverse strand sequences were assembled for each unique haplotype with SeqMan 2 (DNAStar, Madison, WI). Amino acid sequences for each locus were aligned in MAFFT ver. 6.606 [51] using the iterative refinement method (≥1,000 iterations) with consistency and weighted sum-of pairs scores (G-INS-i). The corresponding nucleotide sequence alignments were derived from amino acid alignments in MacClade ver. 4.03 [52]. No indels were inferred for any of the six sequence sets. DNAsp 5.10 (http://www.ub.es/dnasp) was used to determine the number of haplotypes (h), numbers of segregating sites that were synonymous (S_s_) or caused amino acid replacements (S_A_), and the average number of nucleotide differences per site between all sequence pairs (pi) [53]. DNAsp also estimated K, the average number of nucleotide differences between all sequence pairs [54] (equation A3) and among replacement sites (K_A_) and synonymous sites (K_S_) and their ratio (*w* = K_A_/K_S_). The effective recombination rate between adjacent sites (R), the PHI test for recombination and Fu and Li's F* test were also calculated. The *w* ratio was used to infer the action of natural selection from comparative sequence data [41]. If replacements/substitutions are deleterious and therefore subject to purifying selection, K_A_<K_S_ and *w*<1. Alternatively, when replacement substitutions are favored by natural selection K_A_>K_S_ and *w*>1.

### Phylogenetic analyses

Maximum likelihood (ML) analyses of nucleotide characters from each of the molecular data matrices were performed using RAxML ver. 7.0.3 [55]. Because RAxML only implements general time reversible (GTR) Q-matrices for nucleotide characters, more restrictive variants of the GTR matrix were not used when selected by the Akaike Information Criterion. Optimal likelihood trees were searched for using 1,000 independent searches starting from randomized parsimony trees with the GTR-GAMMA model and four discrete rate categories. Likelihood bootstrap (BS) analyses [56] were conducted with 2,000 replicates with ten searches per replicate using the “–f i” option, which “refine[s] the final BS tree under GAMMA and a more exhaustive algorithm” [57].

### Tests for positive selection

CodeML [41] in the PAML package was used to perform a maximum likelihood analysis of protein-coding DNA sequences using codon substitution models [58]. CodeML estimated K_A_ and K_S_ and performed likelihood ratio tests (LRTs) of positive selection along lineages based on *w* to identify amino acid sites potentially under positive selection. We loaded the ML tree topology derived from RAxML and enforced that as a constraint. PAML was set to explicitly account for the nucleotide content at each codon position when calculating K_A_ and K_S_. PAML optimized the branch lengths based on the particular model that it employed for each analysis. Five models (M0, M1a, M2a, M7 and M8) were compared. These five were reported to be the most effective models for detecting positive selection based on both simulations and empirical data [41]. Model M0 assumes and calculates one ω for all codons. Model M1a is a neutral model that assumes two site classes: *w*_0_<1 (estimated empirically from the data) and *w*_1_ = 1. Model M2a is a selection model that is compared with M1a by a LRT. It adds a third site class to M1a, with *w*_2_>1 estimated empirically. Model M7 (beta) is a flexible null model, in which ω for a codon is a random draw from the beta-distribution, with 0<*w*<1. Model M8 (beta & *w*) is compared with M7 (beta) by a LRT. It adds an extra site class to M7 (beta), with *w*_s_>1 estimated empirically. Positive selection was inferred at individual amino acids using the Bayes empirical Bayes method [59].

A star tree is commonly used as a null model in phylogenetic comparative methods. All branches emerge from a single common ancestor (no topology) rather than emerging internally from one another and all branches are of equal length (no differential stasis). All branches in a star tree evolve independently of one another. Recombination among nuclear genes can homogenize haplotypes and thus create a star phylogeny. If a phylogeny derived from a dataset is resolved as a star phylogeny or if the data fit a star phylogeny as well as they fit any alternative phylogeny then the branches are said to evolve independently. Independence is desirable when testing for evidence of selection because specific hierarchies in which branches evolve in a dependent manner may lead to false detection of sites under selection because the hierarchy may be incorrect for sites under selection [59]. Results obtained from the analysis of star trees were highly similar to those obtained from the estimated gene tree (results not shown), suggesting that positive selection results were not seriously affected by possible recombination events.

### Phenotype correlation analysis

Phenotypic data consisted of DENV2 midgut infection barriers (MIB = proportion of orally exposed female mosquitoes that fail to develop a midgut infection) and midgut escape barriers (MEB = proportion of females with a midgut infection that fail to develop a disseminated infection). Phenotypic data were not available for the Thailand or Mindin collections. Pearson correlation coefficients and Fisher exact tests were performed to compare the average number of nucleotide differences per site between all sequence pairs (pi), the average number of nucleotide differences between all sequence pairs (K), among replacement sites (K_A_), among synonymous sites (K_S_) and *w* between each phenotype (i.e. MIB, MEB) using R v2.11.1 (http://cran.r-project.org/).

## Results

### Intraspecific patterns of siRNA and miRNA gene variation in *Ae. aegypti*

**Figure 3:**
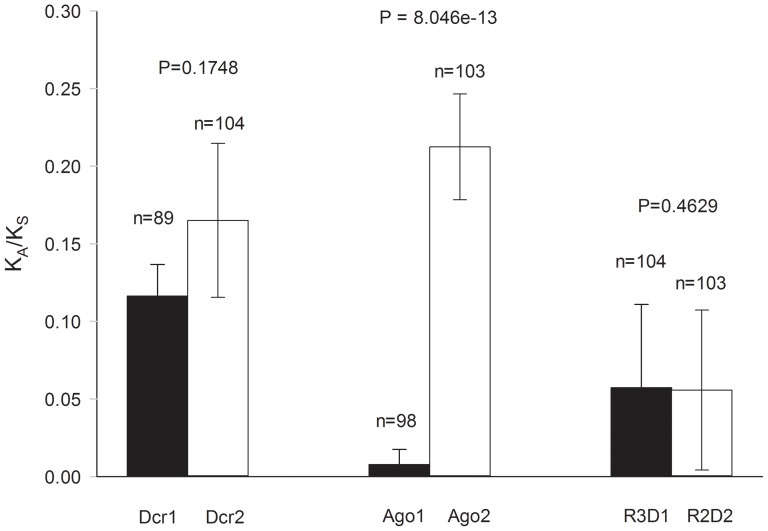
The ratio (*w* = K_a_/K_s_) for miRNA (*ago1*, *dcr1*, and *r3d1*) and siRNA (*ago2*, *dcr2*, and *r2d2*) pathway genes among *Ae. aegypti* collections. The average number of nucleotide differences among replacement sites (K_a_) relative to the average number of nucleotide differences among synonymous sites (K_a_).

Table 2 lists, for each gene and each mosquito collection, sample sizes (N), numbers of segregating sites encoding synonymous (S_s_) and replacement (S_a_) substitutions, numbers of haplotypes (h), average numbers of nucleotide differences per site between all sequence pairs (pi), average numbers of nucleotide differences between all sequence pairs (k), average numbers of nucleotide differences among replacement sites (K_A_), average numbers of nucleotide differences among synonymous sites (K_S_), K_A_/K_S =_ *w*, effective recombination rates between adjacent sites (R), and the PHI tests for recombination alongside Fu and Li's F* test. Graphs of *w* for all six genes appear in Figure 3. In *Ae. aegypti*, *w* was 1.4-fold higher in *dcr2* than in *dcr1*, as compared to 5.4-fold higher in an interspecific study in *Drosophila* [32]. The *Ae. aegypti* ω ratio in *ago2* was 27.4 fold higher than in *ago1* (the K_A_ for *ago1* in all *Drosophila* spp. was zero [32]) . The nearly six-fold higher *w* ratios found in *r2d2* as compared to *r3d1* in *Drosophila* spp. [32] were not found among *Ae. aegypti* collections. Instead *w* was approximately the same in both genes. The probability values from Fisher's exact test of S_a_ and S_s_ appear above the bar graphs for the three gene pairs in Figure 3; only the *ago1* vs. *ago2* comparison in mosquitoes was significantly different. In contrast, among *Drosophila spp*. all three comparisons were significant.

**Table 2:** Intraspecific patterns in miRNA and siRNA pathway genes of *Ae. aegypti*.

Site	N	S_s_	S_a_	h	p	k	K_S_	K_A_	K_A_/K_S_	R	PHI	F*
**Dcr1**												
All Sites	103	158	103	206	0.0068	37.94	0.0212	0.0025	0.1163	0.0125	1.11E-16	0.31
Poza Rica	18	88	44	36	0.0067	40.08	0.0210	0.0022	0.1050	0.0087		1.31
Chetumal	18	84	30	36	0.0045	28.02	0.0148	0.0013	0.0908	0.0036		0.82
L.d.Tejada	18	59	24	36	0.0041	25.46	0.0129	0.0014	0.1053	0.0123		1.13
Thailand	20	67	33	40	0.0041	24.49	0.0121	0.0016	0.1326	0.0051		0.66
PK10	19	54	21	38	0.0042	23.54	0.0126	0.0016	0.1234	0.0062		1.42
Mindin	10	82	43	20	0.0065	40.41	0.0183	0.0026	0.1424	0.0154		0.99
**Dcr2**												
All Sites	104	109	85	190	0.0050	23.87	0.0141	0.0023	0.1650	0.0080	1.63E-14	−0.27
Poza Rica	18	32	24	27	0.0023	10.92	0.0063	0.0010	0.1651	0.0007		0.13
Chetumal	18	20	12	32	0.0021	9.82	0.0060	0.0009	0.1426	0.0072		1.25
L.d.Tejada	18	32	28	33	0.0036	16.98	0.0083	0.0021	0.2503	0.0114		0.82
Thailand	20	26	14	39	0.0028	13.41	0.0088	0.0010	0.1129	0.0090		2.06*
PK10	20	47	25	40	0.0047	22.19	0.0135	0.0019	0.1421	0.0240		1.45
Mindin	10	71	39	20	0.0060	28.44	0.0156	0.0028	0.1772	0.0151		0.31
**Ago1**												
All Sites	104	76	10	136	0.0076	14.91	0.0310	0.0002	0.0077	0.0127	6.42E-13	−0.23
Poza Rica	18	37	1	28	0.0057	13.52	0.0230	0.0001	0.0026	0.0085		1.65*
Chetumal	18	26	0	8	0.0016	3.71	0.0063	0.0000	0.0000	0.0000		0.17
L.d.Tejada	18	29	0	22	0.0040	9.49	0.0162	0.0000	0.0000	0.0050		1.68*
Thailand	20	40	2	38	0.0062	14.68	0.0244	0.0002	0.0094	0.0118		1.87**
PK10	20	37	5	38	0.0074	14.41	0.0277	0.0007	0.0268	0.0480		1.4
Mindin	10	63	4	20	0.0084	19.62	0.0321	0.0002	0.0065	0.1044		0.26
**Ago2**												
All Sites	104	52	75	165	0.0053	13.31	0.0134	0.0029	0.2124	0.0041	2.42E-13	0.97
Poza Rica	18	15	14	30	0.0032	7.86	0.0076	0.0017	0.2224	0.0096		1.36
Chetumal	18	13	18	32	0.0030	7.37	0.0068	0.0017	0.2442	0.0063		0.47
L.d.Tejada	18	12	18	34	0.0034	8.48	0.0071	0.0019	0.2630	0.0164		1.07
Thailand	20	8	11	19	0.0020	4.90	0.0041	0.0012	0.2995	0.0039		0.57
PK10	20	7	9	34	0.0020	5.05	0.0046	0.0012	0.2604	0.0464		1.68*
Mindin	10	33	23	19	0.0076	21.91	0.0186	0.0039	0.2086	0.0063		1.71*
**R3D1**												
All Sites	104	21	9	87	0.0046	4.47	0.0163	0.0009	0.0572	0.0131	2.64E-03	0.69
Poza Rica	18	14	2	17	0.0041	3.98	0.0131	0.0011	0.0861	0.0091		1.23
Chetumal	18	9	2	9	0.0023	2.20	0.0064	0.0009	0.1415	0.0009		0.96
L.d.Tejada	18	13	3	16	0.0040	3.92	0.0152	0.0004	0.0270	0.0075		0.64
Thailand	20	4	0	4	0.0012	1.14	0.0048	0.0000	0.0000	0.0000		1.02
PK10	20	12	4	37	0.0048	4.67	0.0159	0.0012	0.0763	0.1607		1.56
Mindin	10	12	2	16	0.0053	5.20	0.0194	0.0006	0.0325	0.0675		1.33
**R2D2**												
All Sites	104	6	5	19	0.0064	2.81	0.0243	0.0014	0.0557	0.0162	6.65E-01	0.46
Poza Rica	18	3	2	6	0.0012	1.04	0.0037	0.0004	0.1005	0.0000		0.79
Chetumal	18	7	1	16	0.0026	2.32	0.0089	0.0006	0.0683	0.0263		0.21
L.d.Tejada	18	4	1	14	0.0022	2.00	0.0074	0.0006	0.0823	0.0436		1.52
Thailand	20	6	2	8	0.0019	1.73	0.0056	0.0008	0.1441	0.0006		0.97
PK10	20	0	2	3	0.0012	0.51	0.0000	0.0015	0.0000	0.0000		0.69
Mindin	10	10	5	15	0.0066	5.90	0.0198	0.0022	0.1122	0.0290		1.18

Sample size (N), numbers of segregating sites that were synonymous (S_s_) or led to amino acid replacements (S_a_), the number of haplotypes (h), the overall pi(the average number of nucleotide differences per site between two sequences) are listed for each gene and collection. Also listed are the average number of nucleotide differences, k and K among synonymous sites (K_s_) and among replacement sites (K_a_) and their ratio (K_a_/K_s_). The effective recombination rate between adjacent sites (R) and the PHI test for recombination are listed alongside Fu and Li's F* test and associated significance tests (*P<0.05, **P<0.01).

Obbard *et al.* [32] found no replacement substitutions in *ago1* among *Drosophila* spp. In contrast, we found 10 nucleotide substitutions that caused amino acid replacements in *Ae. aegypti ago1*. In *ago2*, ω was 2.5 fold higher among *Drosophila* spp. than among *Ae. aegypti* populations. In *dcr1*, *w* was approximately the same among *Drosophila* spp. as among *Ae. aegypti* populations, while *w* in *dcr2* was 3.5 fold higher among *Drosophila* spp. than among *Ae. aegypti* populations. In *r3d1*, *w* was 2.2 fold higher among *Drosophila* spp. than among *Ae. aegypti* populations while in *r2d2*, ω was 13.2 fold higher among *Drosophila* spp. The same trends in *w* occurred in all six *Ae. aegypti* collections (Table 2).

While not significant, comparison of *w* between Dcr1 and Dcr2 among *Ae. aegypti* populations reveals the same trend as among *Drosophila* spp, with a higher ω in the siRNA pathway gene. In contrast, replacement substitutions in *ago1* were detected in four of the six *Ae. aegypti* collections while no replacement substitutions were found among four species of *Drosophila*. The same large disparity in ω between *Drosophila* Ago1 and Ago2 was evident among *Ae. aegypti* populations, but the same was not true of the *Ae. aegypti* dsRNA-binding proteins R3D1 and R2D2, amongst which *w* was approximately identical.

### Functional domain analysis

**Figure 4:**
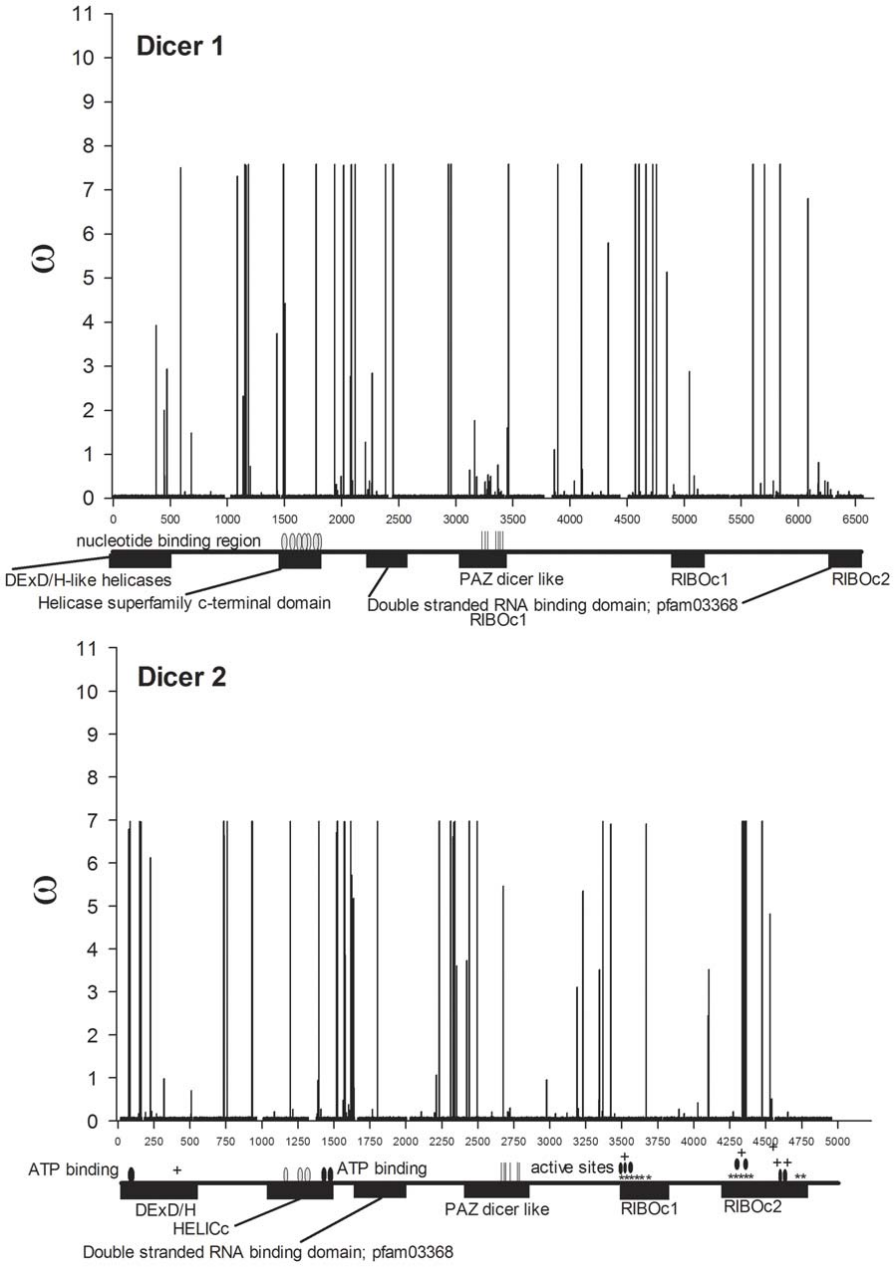
*w* ratios for all nucleotides mapped across annotated Dicer genes. The amino-terminal domains of most Dicer enzymes contain a DExH-box RNA or ERCC4-like helicase domain followed by members of helicase superfamily c-terminal domains containing a number of nucleotide binding regions (gray ovals) and an ATP binding domain on Dcr 2 (black ovals). One or two double stranded RNA-binding domains (dsRBDs) consisting of ∼100 amino acid residues occur in the center and the carboxyl end of Dicers. The first of these dsRBDs is followed by the oligonucleotide-binding (indicated with vertical lines) PAZ domain located in the center of Dicer where it binds the 5′ phosphates and 2 nt 3′ hydroxyl overhangs. Cleavage is accomplished by dimerized RNase III domains (labeled RIBOc). Active sites (black ovals) are shown on Dcr2. The areas of dimerization are labeled with ‘*’ while regions of metal ion-binding are labeled with a ‘+.’

**Figure 5:**
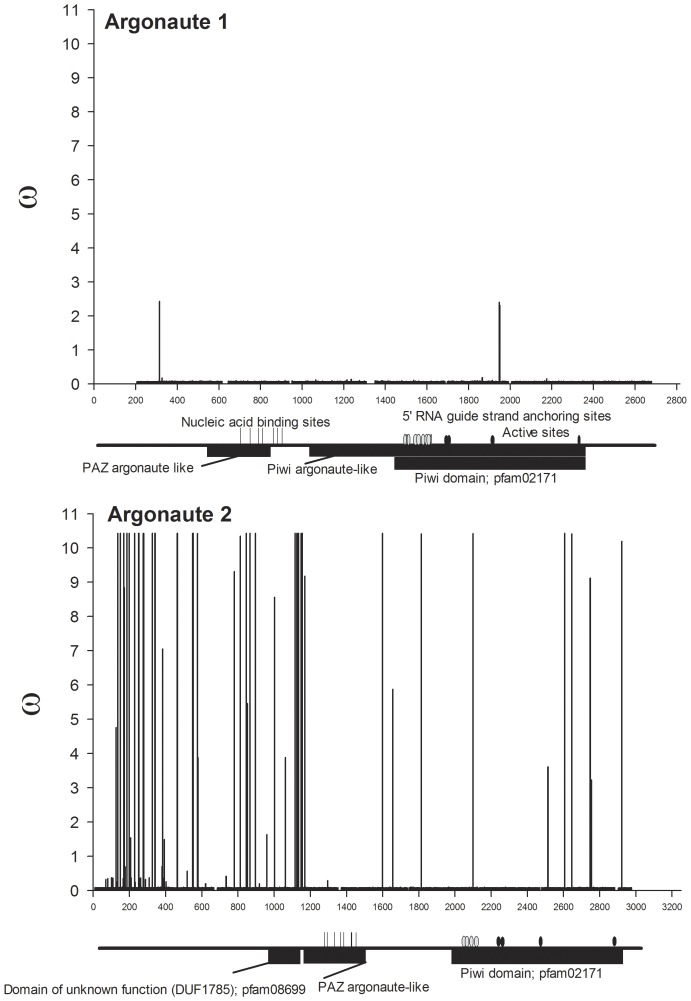
*w* ratios for all nucleotides mapped across annotated Argonaute genes. Eukaryotic argonaute proteins are characterized by having both PAZ and Piwi domains. Piwi domains are found only in Ago proteins and are structurally related to the RNase H family of ribonucleases. Nucleic acid binding sites are indicated by vertical lines. The 5′ ends of siRNAs and miRNAs are important for mRNA target recognition and definition of the site of RNA cleavage. These binding sites are indicated by grey ovals. Active sites (black ovals) in the Ago1 ribonuclease correspond to Tyr684, Glu686, Pro757 and Gly895 and in Ago 2 correspond to Asp740, His742, Asp812, and His950.

**Figure 6:**
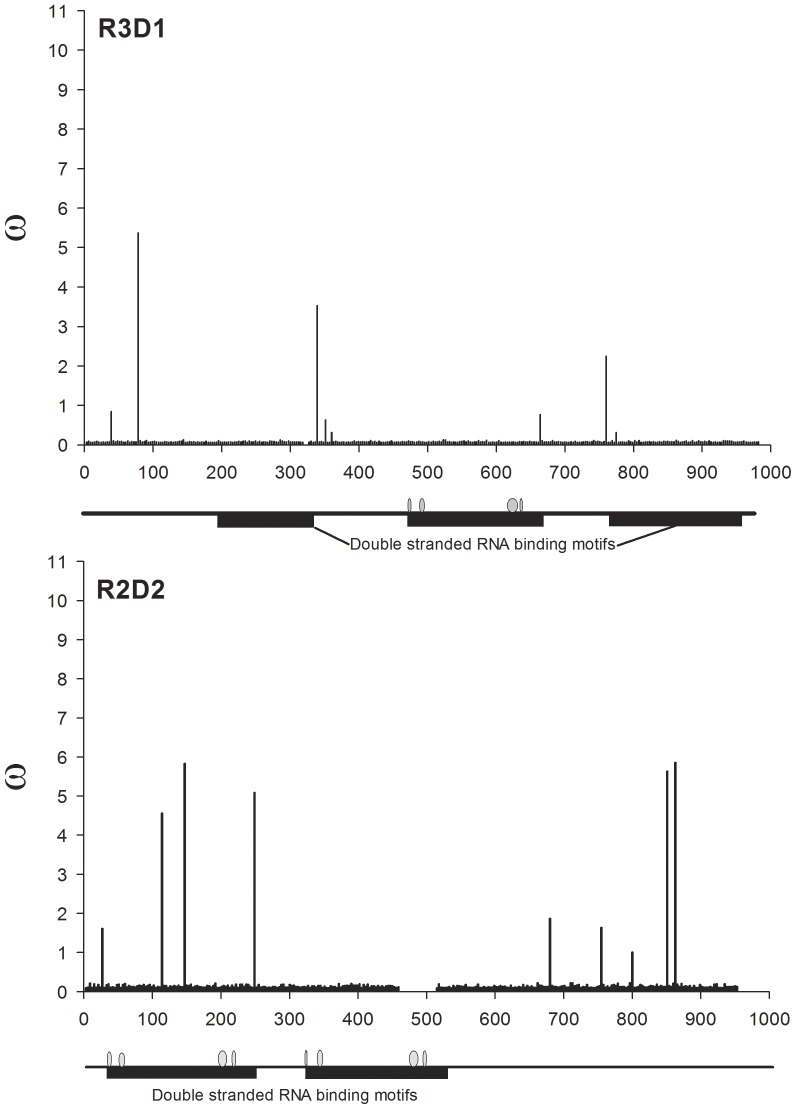
*w* ratios for all nucleotides mapped across annotated R3D1 (cognate binding protein for Dcr1) and R2D2 (binding protein for Dcr2).

Functional domains on each of the six proteins were defined by annotations in GenBank: Dcr1: AAW48724 and Dcr2: AAW48725 (DExD/H-like helicases, helicase superfamily c-terminal domains, dsRNA-binding, PAZ dicer-like, and ribonuclease domains); Ago1: XP_001662554 and Ago2: ACR56327 (conserved domains of unknown function (DUF), PAZ argonaute-like and PIWI domains); R3D1: XP_001659426 and R2D2: XP_001655660 (double-stranded RNA-binding motifs) (Figures 4–6 and Table 3). The average numbers of nucleotide differences per site between all sequence pairs (pi) were compared for each gene. The proportion of segregating sites in regions of known function versus regions where no function has been assigned to date were not significantly different for any of the six genes as determined by Fisher's Exact Test. Average values of pi were compared between regions of known versus unassigned function using a Student's t-test and a significant difference was only seen for R3D1 in which pi was greater in regions of known function (Table 3).

**Table 3:** Functional domain analysis.

Gene	Function	Sites	SNPs	Prob.	pi	pi(known function)	Prob.
				FET		pi(unassigned)	t-test
Dcr1	Unassigned	39	0		0.0000		
	DExD/H-like helicase	474	11		0.0109		
	Unassigned	972	50		0.0102		
	Helicase superfamily c-terminal domain	327	9		0.0030		
	Unassigned	465	32		0.0085		
	Double stranded RNA binding domain; pfam03368	288	10		0.0063		
	Unassigned	513	9		0.0030		
	PAZ_dicer_like	360	28		0.0070		
	Unassigned	1524	39		0.0049		
	RIBOc1	255	3		0.0050		
	Unassigned	633	35		0.0099	0.0063	
	RIBOc2	333	19	0.9448	0.0056	0.0061	0.9109
Dcr2	Unassigned	63	6		0.0126		
	DExD/H-like helicase	426	28		0.0054		
	Unassigned	519	14		0.0048		
	HELICc	321	13		0.0029		
	Unassigned	208	24		0.0074		
	Double stranded RNA binding domain; pfam03368	266	10		0.0046		
	Unassigned	483	23		0.0081		
	PAZ	351	13		0.0058		
	Unassigned	684	25		0.0055		
	RIBOc1	246	3		0.0006		
	Unassigned	465	12		0.0042		
	RIBOc2	485	15		0.0046	0.0040	
	Unassigned	229	8	0.6061	0.0024	0.0064	0.1322
Ago1	Domain of Unknown Function DUF1785; Pfam 08699	159	0		0.0000		
	PAZ_argonaute_like	273	3		0.0015		
	Piwi_ago-like	1257	62		0.0081		
	Piwi domain; pfam02171	885	41		0.0088		
	5′ RNA guide strand anchoring site	12	1		0.0008	0.0038	
	Unassigned	204	8	1.0000	0.0093	0.0093	-
Ago2	Unassigned	933	22		0.0059		
	Domain of Unknown Function DUF1785; Pfam 08699	147	6		0.0087		
	PAZ_argonaute_like	312	12		0.0069		
	Unassigned	513	18		0.0081		
	Piwi domain; pfam02171	912	14		0.0027	0.0061	
	Unassigned	78	2	0.1482	0.0014	0.0052	0.7332
R3D1	Unassigned	195	4		0.0036		
	Double-stranded RNA binding motif	135	3		0.0069		
	Unassigned	138	6		0.0010		
	Double-stranded RNA binding motif	201	8		0.0061		
	Unassigned	96	2		0.0030		
	Double-stranded RNA binding motif	198	6		0.0061	0.0063	
	Unassigned	15	0	0.7084	0.0000	0.0019	0.0099
R2D2	Unassigned	30	2		0.0010		
	Double-stranded RNA binding motif	210	4		0.0009		
	Unassigned	66	3		0.0155		
	Double-stranded RNA binding motif	153	1		0.0014	0.0011	
	Unassigned	441	11	0.1775	0.0064	0.0076	0.2665

The proportions of segregating sites in regions of known function versus regions where functions have not been assigned were compared by Fisher's Exact Test (FET) and the resulting probability is listed. Average values of pi(average number of nucleotide differences per site between sequence pairs) were compared between regions of known versus unassigned function using a student's t-test and a significant difference was only seen for R3D1, in which pi was greater in regions of known function.

### Tests for positive selection

Maximum likelihood analysis of protein-coding DNA sequences was used to estimate the average number of nucleotide differences among replacement sites (K_A_) and synonymous sites (K_S_) and perform LRTs for positive selection. The ML tree topologies derived from RAxML were enforced as a constraint in CodeML from the PAML package [41]. Five models (M0, M1a, M2a, M7 and M8) were compared and results for each of the five models and each of the six genes are shown in Table S1. For each gene the model with the greatest likelihood score is highlighted in grey. In each case the M2a model had a significantly better fit than the M1a model and the M8 model had a significantly better fit than the M7 model, implying that models of positive selection had a better fit than neutral models. All of the amino acids identified using the naïve empirical Bayes (NEB) method were also identified using the Bayes empirical Bayes (BEB) method, while a few additional sites were identified with BEB.

The alternative amino acids found to be under positive selection using BEB are listed in Table S2 alongside *w* from the M8 model and its standard error. All replacement to synonymous substitution ratios (*w*) are mapped across *dicer* genes in Figure 4, across *argonaute* genes in Figure 5 and across genes encoding dsRNA-binding proteins in Figure 6. Even though clusters are visually evident, the distances between positively selected sites (PSSs) were formally subjected to a chi-square goodness-of-fit test to determine if they were distributed in a random (Poisson) fashion. Only *ago2*, *dcr1*, and *dcr2* had enough sites to perform this test and the PSSs all three of these genes were clustered rather than randomly distributed (analyses available on request).

An additional test was conducted on PSSs using the program Sorting Intolerant from Tolerant (SIFT) [42] to determine whether the amino acid changes in Table S2 are predicted to affect protein function. SIFT scores replacement substitutions on a scale from 0–1, where scores at or below 0.05 are likely to change protein function, whereas higher scores are not. Thirty PSSs were detected in Dcr1, eight (27%) of which were likely to change protein function; in Dcr2, 16 of 38 (42%) PSSs were likely to change protein function (Figure 4). In Dcr1, a single cluster consisting of positively-selected sites 384, 385, 388 and 395 was detected in a region without assigned function between the DExD/H-like helicase and helicase superfamily C-terminal domains. Replacements at PSSs 497, 502 and 592 were in the helicase superfamily C-terminal domain and two of these were predicted to change function. Replacements at PSSs 795, 817 and 978 were in the dsRNA-binding domain but were not predicted to change function. In Dcr2, one cluster of PSSs occurred in the ribonuclease III carboxyl-terminal domain and included amino acid sites 1446, 1450, and 1454, which are among the residues responsible for dimerization. Missense mutations in an RNase III-encoding domain of *Drosophila dcr2* resulted in a profound loss of dsRNA processing activity and destabilization of the protein [24]. A second Dcr2 PSS cluster consisted of 8 PSSs, six with significant SIFT scores, in a region of unassigned function between the ribonuclease III C terminal domain and the dsRNA binding domain. A cluster also occurred in a region of unassigned function amino-terminal to the PAZ domain.

Replacement substitutions in *ago1* were detected in four of the six *Ae. aegypti* collections even though no replacement substitutions were found in *ago1* among four species of *Drosophila*. It is unclear why diversifying selection would occur within and among collections of *Ae. aegypti* while only purifying selection is evident among *Drosophila spp.* Three PSSs were identified in Ago1 but none of these was predicted to change protein function. In strong contrast, 16 of 38 PSSs were likely to change protein function in Ago2 (Figure 5). Four clusters of PSSs were found in Ago2; one of the clusters occurred in the amino-terminal region of the PAZ domain at residues 372, 375, 378, 383, 385, and 390, and the 383 and 390 replacements had significant SIFT scores. However, the oligonucleotide binding domain of PAZ occurs in the carboxyl-terminus in residues 418–472 according to GenBank annotation ACR56327. The amino-terminus of *ago2* encodes a series of poly-glutamines, however the function(s) of these residues are unknown. One intriguing PSS occurred in the 5′ RNA guide strand anchoring site in the amino-end of the Piwi domain. This corresponds to amino acid residue 700 and involves a threonine-methionine replacement. Of two PSSs identified in R3D1, one was likely to change protein function. In R2D2, four of five PSSs were predicted to alter function.

### Phenotype correlation analysis

Phenotype correlation analysis was performed to identify potential correlations of mosquitoes with DENV2 midgut infection barrier (MIB) and escape barrier (MEB) phenotypes with the number of nucleotide differences between all sequence pairs (k), nucleotide differences per site between all sequence pairs (pi) and and/or the number of nucleotide differences among replacement sites (K_A_) and among synonymous sites (K_S_) and/or the K_A_/K_S_ ratio (*w*). Phenotype data were available for Poza Rica (MIB: 21%, MEB: 18%), Lerdo de Tejada (MIB: 25%, MEB: 36%), and Chetumal, Mexico (MIB: 9%, MEB: 8%) [43],[44] and PK10, Senegal (MIB: 8%, MEB: 76%) [45] collections. Pearson correlation coefficients are displayed in Table 4. No significant MIB correlations were found. No significant MEB correlations with these variables were observed for the *argonaute*, *r3d1* or *r2d2* genes. However, *w* in *dcr1* was significantly positively correlated with MEB and pi, k, and K_S_ were significantly correlated with MEB for *dcr2*. This suggests the possibility that diversity in Dicers may increase the frequency of mosquitoes with MEBs.

**Table 4:** Phenotype correlation analysis.

Gene	p	k	Ks	Ka	Ka/Ks
Ago1					
MIB	0.000	0.031	0.003	0.343	0.338
MEB	0.629	0.439	0.564	0.837	0.832
Ago2					
MIB	0.640	0.646	0.489	0.580	0.019
MEB	0.585	0.580	0.773	0.581	0.398
Dcr1					
MIB	0.108	0.152	0.105	0.085	0.017
MEB	0.203	0.338	0.304	0.020	0.900*
Dcr2					
MIB	0.026	0.026	0.157	0.064	0.723
MEB	0.956*	0.957*	0.984**	0.608	0.002
R3D1					
MIB	0.050	0.051	0.153	0.386	0.493
MEB	0.642	0.640	0.574	0.066	0.216
R2D2					
MIB	0.000	0.041	0.072	0.410	0.543
MEB	0.299	0.520	0.633	0.829	0.718

To identify potential correlations of Ae aegypti susceptibility to dengue virus infection with measures of nucleotide and amino acid diversity in RNAi genes, Pearson correlation analyses were performed between DENV-2 MIB (the proportion of orally virus-exposed female mosquitoes that fail to develop a midgut infection) and MEB (the proportion of females with a midgut infection that fail to develop a disseminated infection) with p (number of nucleotide differences per site between all sequence pairs), k (number of nucleotide differences between all sequence pairs), K_A_ (number of nucleotide differences among replacement sites), K_S_ (number of nucleotide differences among synonymous sites) and K_A_/K_S_. Pearson correlation coefficients are shown.

* P≤0.05,

** P≤0.01.

## Discussion

### Intraspecific patterns of variation between miRNA and siRNA pathway genes in *Ae. aegypti*

In contrast to *Drosophila* spp., we found that in *Ae. aegypti* mosquitoes both exo-siRNA and miRNA pathway genes appear to be undergoing rapid, positive, diversifying selection. However, in similarity to *Drosophila*, most positively selected sites occurred in protein regions without defined functions (Table S2 and Figs. 3–5). Our observations are consistent with a hypothesis that diversifying selection acts on both *dcr1* and *dcr2* to maintain the intraspecific *w* ratios among *dcr1* genes of *Ae. aegypti* populations at the same level as among *Drosophila* species. Mandibulate arthropods are unique in having two Dicers [34]. With the exception of Cnidarians and Porifera, the remainder of animals so far examined, including vertebrates, possess a single Dicer that produces miRNAs and, in the case of invertebrates such as *C. elegans*, also siRNAs. It is possible that Dcr1 retains some role in antiviral RNAi in mosquitoes but not in *Drosophila*. Mosquitoes (Culicidae) are members of the primitive fly suborder Nematocera while Drosophilidae are one of the most recently evolved of fly families [60]. In addition, replication of some mammalian viruses has been shown to be indirectly inhibited by host miRNAs and some viruses exploit host miRNAs during replication [61]; however, potential roles of mosquito miRNAs in arbovirus replication have not been explored. It is possible that components that have been assigned functions in distinct RNA silencing pathways, including the miRNA pathway, interact with or serve as redundant or backup antiviral mechanisms for the exo-siRNA pathway in insects. Evidence of interactions between components of RNA silencing pathways in *Drosophila* was provided by the demonstration that R3D1-Dcr2 heterodimers, rather than the canonical R2D2-Dcr2 complexes, are involved in Ago2-RISC-loading of endo-siRNAs [5], [6]. The endo-siRNA pathway, which has been shown to function in suppressing transposon activity in somatic cells of *Drosophila*, can also be triggered by transient transfection of exogenous dsRNA, suggesting a potential role in antiviral defense [62]. Potential interactions between siRNA and miRNA pathways in mosquitoes, particularly in antiviral defense or control of transposon activity, remain to be examined.

Evidence for a role of the piRNA pathway in insect antiviral defense also has emerged recently. In our examination of antiviral RNAi in *Anopheles gambiae* mosquitoes we found that dsRNA-mediated silencing of the *ago3* gene concurrent with O'nyong-nyong virus infection resulted in increased virus titers, hinting at a possible role for Ago3 in antiviral immunity [25]. Furthermore, RNA virus-specific piRNAs, in addition to viral siRNAs, were recently described in *Drosophila* ovary cells [63] and other studies showed that *piwi*-family mutants of *Drosophila* were more susceptible to *Drosophila* virus X infection than wild-type flies [8]. We also found that in cultured C6/36 (*Aedes albopictus*) cells a single-nucleotide deletion in *dcr2* causes a defect in the exo-siRNA pathway-mediated antiviral defense that was apparently compensated by the piRNA pathway [30], [64]. The redundant role of the piRNA pathway in antiviral defense in mosquito somatic cells and its particular relevance in *dcr2*-null cell culture lines has recently been confirmed [65].

### Sources of diversifying selection

Obbard et al. [34] suggested that a “molecular arms race” with pathogenic virus suppressors of RNAi drives siRNA pathway gene diversity in *Drosophila* spp. There are various reasons to believe that infections with arboviruses are not the drivers of diversifying selection that we have documented in the miRNA and siRNA pathways in *Ae aegypti*. A hallmark of arboviruses is that they have little, if any, effect on survivorship or fecundity in their insect hosts [66]. Two studies further suggest that selection has, in fact, prevented the evolution of potent VSRs in arboviruses [28], [38]. Even if infection by arboviruses imposed a minimal fitness effect, a number of field studies have demonstrated that very few mosquitoes (10^−3^ to 10^−4^) collected in areas endemic for certain arboviruses are actually infected at any given time [67], [68], [69] thus providing only rare opportunities for selection. An interesting alternative possibility for selection may involve the recently discovered high prevalence of persistent insect-only flaviviruses in natural populations [70], [71], [72]. These viruses are maintained through vertical transmission from one generation to the next without obvious pathogenesis and without requiring horizontal transmission through infected vertebrates.

We suggest that infections with entomopathogenic viruses or transposon invasion and movement are more likely causes of the diversifying selection detected in this study. Unfortunately, few mosquito-pathogenic RNA viruses have been identified or well-studied. One such virus, Nodamura virus (*Nodaviridae*), was isolated from *Culex tritaeniorhynchus* in Japan [73] and can experimentally infect and produce pathogenesis in *Ae. aegypti* [74]. It has a bipartite, positive-strand RNA genome that replicates through a dsRNA intermediate. Unique features of viruses in this family are that their replication complexes are sequestered in membrane-enclosed spherules within the mitochondrial outer membrane during replication and they encode B2-type VSRs [75]. Small RNAs that appeared to be derived from the RNA genome of a previously-undescribed nodavirus were recently discovered by deep sequencing analysis of a small RNA library from *Ae. aegypti* [63], suggesting that other potentially pathogenic mosquito viruses remain to be found.

### Phenotype correlation analysis

Our *a priori* hypothesis was that *Ae. aegypti* collections that were more refractory for DENV2 disseminated infection would also have higher rates of evolution in genes encoding components of the siRNA pathway compared to DENV2 susceptible populations. The trends shown in correlation of vector competence with certain measures of genetic diversity in RNAi pathway genes in Table 4 need to be tested in more *Ae. aegypti* populations and possibly in other *Aedes* species. Furthermore, we need to perform quantitative trait loci mapping and association studies to test for a correlation between miRNA and siRNA pathway alleles and arbovirus susceptibility. If a correlation is detected it could suggest that RNA silencing evolved in mosquitoes as a means to combat entomopathogenic virus infection or genome invasion by transposons but that this evolution may have indirectly provided a regulatory mechanism for replication and transmission of arboviruses.

## Supporting Information

Table S1 **CodeML results for each of the five models for detection of positive selection and each of the six genes.** For each gene, the ML model is highlighted in grey. The number of positively selected sites (PSS) identified using the naive empirical Bayes (NEB) and Bayes empirical Bayes (BEB) methods are listed for each gene. l = −log likelihood ratio. The likelihood ratio test was computed between Models M2a and M1a (**c^2^_[1 d.f.]_ = 2**ΔL) and between Models M7 and M8 (χ^2^ **_[1 d.f.]_ = 2**ΔL) in all 12 comparisons, P<0.0001.
(DOCX)

Table S2 **Alternative amino acids found to be under positive selection in CodeML using BEB.** The locations of the replacements are indicated in parentheses (U = region with unassigned function, Pw = Piwi, Pz = PAZ, DUF = domain of unknown function, Hc = Helicase superfamily c-terminal domain, Hcd = Helicase dimerization domain, Dc = DExD/H-like helicase, Db = double stranded RNA binding domain). Sites are listed alongside w ratios from the M8 model and its standard error. SIFT scores <0.05 indicate the replacement amino acid is likely to change protein function and are underlined and appear in bold. Sites that are clustered (<10 nt apart) appear in boxes.
(DOCX)
