# Supplementary material for: Rapid Intraspecific Evolution of miRNA and siRNA Genes in the Mosquito Aedes aegypti
Source: PLoS One. 2012 Sep 21;7(9):e44198. doi: 10.1371/journal.pone.0044198 (PMC3448618; doi:10.1371/journal.pone.0044198)
Supplement: Table S1 — CodeML results for each of the five models for detection of positive selection and each of the six genes. For each gene, the ML model is highlighted in grey. The number of positively selected sites (PSS) identified using the naive empirical Bayes (NEB) and Bayes empirical Bayes (BEB) methods are listed for each gene. l = −log likelihood ratio. The likelihood ratio test was computed between Models M2a and M1a (c2[1 d.f.] = 2ΔL) and between Models M7 and M8 (χ2 [1 d.f.] = 2ΔL) in all 12 comparisons, P<0.0001. (DOCX) [file pone.0044198.s001.docx]

**Table S1:** CodeML results for each of the five models for detection of positive selection and each of the six genes. For each gene, the ML model is highlighted in grey. The number of positively selected sites (PSS) identified using the naive empirical Bayes (NEB) and Bayes empirical Bayes (BEB) methods are listed for each gene. l = - log likelihood ratio. The likelihood ratio test was computed between Models M2a and M1a (**χ^2^_[1 d.f.]_ = 2Δ**l ) and between Models M7 and M8 (**χ^2^_[1 d.f.]_ = 2Δ**l). In all 12 comparisons, P < 0.0001.

Model(parameters) l p_0_ p_1_ p_2_ p_3_ p_4_ p_5_ p_6_ p_7_ p_8_ p_9_ p_10_

w_0_ w_1_ w_2_ w_3_ w_4_ w_5_ w_6_ w_7_ w_8_ w_9_ w_10_

Dicer 1

M0(1) -24789.1

M1a(neutral)(2) -23574.7 0.9486 0.0514

0.0017 1.0000

M2a(selection)(4) -23148.4 0.9445 0.0434 0.0121

**PSS:NEB=25;BEB=27** 0.0012 1.0000 8.7917

**χ^2^_[1 d.f.]_ = 2Δ**l **=** 852.70

M7(beta)(10) -23599.1 0.1000 0.1000 0.1000 0.1000 0.1000 0.1000 0.1000 0.1000 0.1000 0.1000

0.0000 0.0000 0.0000 0.0000 0.0000 0.0000 0.0000 0.0000 0.0000 1.0000

M8(beta&w)(11) -23179.3 0.0988 0.0988 0.0988 0.0988 0.0988 0.0988 0.0988 0.0988 0.0988 0.0988 0.0120

**PSS:NEB=25;BEB=30** 0.0000 0.0000 0.0000 0.0000 0.0000 0.0000 0.0000 0.0000 0.0000 0.9075 9.0542

**χ^2^_[2 d.f.]_ = 2Δ**l **=** 839.54

Dicer 2

M0(1) -14572.6

M1a(neutral)(2) -14030.0 0.9309 0.0691

0.0010 1.0000

M2a(selection)(4) -13812.3 0.9351 0.0466 0.0183

**PSS:NEB=29;BEB=33** 0.0056 1.0000 8.2708

**χ^2^_[1 d.f.]_ = 2Δ**l **=** 435.44

M7(beta)(10) -14035.9 0.1000 0.1000 0.1000 0.1000 0.1000 0.1000 0.1000 0.1000 0.1000 0.1000

0.0000 0.0000 0.0000 0.0000 0.0000 0.0000 0.0000 0.0000 0.0000 1.0000

M8(beta&w)(11) -13821.0 0.0978 0.0978 0.0978 0.0978 0.0978 0.0978 0.0978 0.0978 0.0978 0.0978 0.0222

**PSS:NEB=32;BEB=39** 0.0000 0.0000 0.0000 0.0000 0.0000 0.0000 0.0000 0.0000 0.0000 0.4921 7.5031

**χ^2^_[2 d.f.]_ = 2Δ**l **=** 429.83

Model(parameters) l p_0_ p_1_ p_2_ p_3_ p_4_ p_5_ p_6_ p_7_ p_8_ p_9_ p_10_

w_0_ w_1_ w_2_ w_3_ w_4_ w_5_ w_6_ w_7_ w_8_ w_9_ w_10_

Argonaute 1

M0(1) -8233.1

M1a(neutral)(2) -8162.6 0.9951 0.0049

0.0038 1.0000

M2a(selection)(4) -8153.6 0.9961 0.0000 0.0039

**PSS:NEB=3;BEB=3** 0.0041 1.0000 2.5363

**χ^2^_[1 d.f.]_ = 2Δ**l **=** 18.00

M7(beta)(10) -8191.4 0.1000 0.1000 0.1000 0.1000 0.1000 0.1000 0.1000 0.1000 0.1000 0.1000

0.0000 0.0000 0.0000 0.0000 0.0000 0.0000 0.0000 0.0000 0.0000 0.1753

M8(beta&w)(11) -8154.2 0.0996 0.0996 0.0996 0.0996 0.0996 0.0996 0.0996 0.0996 0.0996 0.0996 0.0039

**PSS:NEB=3;BEB=3** 0.0000 0.0001 0.0003 0.0007 0.0013 0.0023 0.0036 0.0057 0.0092 0.0176 2.5365

**χ^2^_[2 d.f.]_ = 2Δ**l **=** 74.44

Argonaute 2

M0(1) -8926.9

M1a(neutral)(2) -8511.2 0.9073 0.0927

0.0000 1.0000

M2a(selection)(4) -8220.9 0.8911 0.0778 0.0311

**PSS:NEB=29;BEB=33** 0.0000 1.0000 12.9915

**χ^2^_[1 d.f.]_ = 2Δ**l **=** 580.61

M7(beta)(10) -8510.4 0.1000 0.1000 0.1000 0.1000 0.1000 0.1000 0.1000 0.1000 0.1000 0.1000

0.0000 0.0000 0.0000 0.0000 0.0000 0.0000 0.0000 0.0000 0.0000 1.0000

M8(beta&w)(11) -8219.4 0.0969 0.0969 0.0969 0.0969 0.0969 0.0969 0.0969 0.0969 0.0969 0.0969 0.0306

**PSS:NEB=29;BEB=38** 0.0000 0.0000 0.0000 0.0000 0.0000 0.0000 0.0000 0.0000 0.0000 1.0000 13.4267

**χ^2^_[2 d.f.]_ = 2Δ**l **=** 582.13

Model(parameters) l p_0_ p_1_ p_2_ p_3_ p_4_ p_5_ p_6_ p_7_ p_8_ p_9_ p_10_

w_0_ w_1_ w_2_ w_3_ w_4_ w_5_ w_6_ w_7_ w_8_ w_9_ w_10_

R3D1

M0(1) -2207.2

M1a(neutral)(2) -2179.1 0.9605 0.0395

0.0000 1.0000

M2a(selection)(4) -2172.4 0.9612 0.0356 0.0032

**PSS:NEB=1;BEB=1**  0.0000 1.0000 12.1247

**χ^2^_[1 d.f.]_ = 2Δ**l **=** 13.32

M7(beta)(10) -2184.6 0.1000 0.1000 0.1000 0.1000 0.1000 0.1000 0.1000 0.1000 0.1000 0.1000

0.0000 0.0000 0.0000 0.0000 0.0000 0.0000 0.0000 0.0000 0.0000 0.8835

M8(beta&w)(11) -2172.7 0.0997 0.0997 0.0997 0.0997 0.0997 0.0997 0.0997 0.0997 0.0997 0.0997 0.0032

**PSS:NEB=1;BEB=2**  0.0000 0.0000 0.0000 0.0000 0.0000 0.0000 0.0000 0.0000 0.0000 0.3840 12.0062

**χ^2^_[2 d.f.]_ = 2Δ**l **=** 23.83

R2D2

M0(1) -1657.0

M1a(neutral)(2) -1639.4 0.9101 0.0899

0.0000 1.0000

M2a(selection)(4) -1632.2 0.9202 0.0688 0.0111

**PSS:NEB=3;BEB=5** 0.0000 1.0000 11.2220

**χ^2^_[1 d.f.]_ = 2Δ**l **=** 14.50

M7(beta)(10) -1642.6 0.1000 0.1000 0.1000 0.1000 0.1000 0.1000 0.1000 0.1000 0.1000 0.1000

0.0000 0.0000 0.0000 0.0000 0.0000 0.0000 0.0000 0.0000 1.0000 1.0000

M8(beta&w)(11) -1632.4 0.0989 0.0989 0.0989 0.0989 0.0989 0.0989 0.0989 0.0989 0.0989 0.0989 0.0111

**PSS:NEB=3;BEB=5** 0.0000 0.0000 0.0000 0.0000 0.0000 0.0000 0.0000 0.0000 0.0000 0.7948 11.5543

**χ^2^_[2 d.f.]_ = 2Δ**l **=** 20.29
