# Supplementary material for: Rapid Intraspecific Evolution of miRNA and siRNA Genes in the Mosquito Aedes aegypti
Source: PLoS One. 2012 Sep 21;7(9):e44198. doi: 10.1371/journal.pone.0044198 (PMC3448618; doi:10.1371/journal.pone.0044198)
Supplement: Table S2 — Alternative amino acids found to be under positive selection in CodeML using BEB. The locations of the replacements are indicated in parentheses (U = region with unassigned function, Pw = Piwi, Pz = PAZ, DUF = domain of unknown function, Hc = Helicase superfamily c-terminal domain, Hcd = Helicase dimerization domain, Dc = DExD/H-like helicase, Db = double stranded RNA binding domain). Sites are listed alongside w ratios from the M8 model and its standard error. SIFT scores <0.05 indicate the replacement amino acid is likely to change protein function and are underlined and appear in bold. Sites that are clustered (<10 nt apart) appear in boxes. (DOCX) [file pone.0044198.s002.docx]

**Table S2. Alternative amino acids found to be under positive selection in CodeML using BEB.**  The locations of the replacements are indicated in parentheses (U = region with unassigned function, Pw= Piwi, Pz=PAZ, DUF=domain of unknown function, Hc =Helicase superfamily c-terminal domain, Hcd = Helicase dimerization domain, Dc= DExD/H-like helicase, Db=double stranded RNA binding domain). Sites are listed alongside ω ratios from the M8 model and its standard error. SIFT scores <0.05 indicate the replacement amino acid is likely to change protein function and are underlined and appear in bold. Sites that are clustered (< 10 nt apart) appear in boxes.

| Codon | AA | BEB(pr. w>1) | w(bar) | +/- | SIFT |  |  |
| --- | --- | --- | --- | --- | --- | --- | --- |
| **Ago1** |  |  |  |  |  |  |  |
| 105 | M,T(U) | 0.998** | 2.430 | 0.659 | 0.07 |  |  |
| 649 | G,S(Pw) | 0.982* | 2.405 | 0.690 | 0.08 |  |  |
| 650 | S,T(Pw) | 0.933 | 2.304 | 0.762 | 0.08 |  |  |
| **Ago2** |  |  |  |  |  |  |  |
| 45 | Q,R,W(U) | 1.000** | 10.428 | 0.260 | R(0.28) | W(0.05) |  |
| 50 | E,G(U) | 1.000** | 10.428 | 0.259 | 0.10 |  |  |
| 56 | Q,E(U) | 1.000** | 10.428 | 0.259 | **0.05** |  |  |
| 57 | Q,R(U) | 0.838 | 8.832 | 3.628 | 0.12 |  |  |
| 62 | Q,R(U) | 1.000** | 10.426 | 0.298 | **0.05** |  |  |
| 66 | Q,E(U) | 1.000** | 10.428 | 0.259 | 0.10 |  |  |
| 76 | Q,H(U) | 1.000** | 10.428 | 0.259 | 0.67 |  |  |
| 84 | Q,K(U) | 1.000** | 10.428 | 0.259 | 0.75 |  |  |
| 92 | Q,D,H(U) | 1.000** | 10.428 | 0.259 | H(0.22) | Q(0.62) |  |
| 93 | Q,H(U) | 0.999** | 10.421 | 0.370 | **0.05** |  |  |
| 109 | L,F(U) | 1.000** | 10.428 | 0.262 | 0.49 |  |  |
| 114 | Q,R(U) | 1.000** | 10.428 | 0.259 | 0.12 |  |  |
| 128 | S,C(U) | 0.661 | 7.049 | 4.716 | **0.02** |  |  |
| 155 | K,E(U) | 1.000** | 10.428 | 0.259 | 0.07 |  |  |
| 183 | R,K(U) | 1.000** | 10.428 | 0.259 | 0.41 |  |  |
| 184 | A,P(U) | 1.000** | 10.428 | 0.259 | 0.35 |  |  |
| 192 | I,V(U) | 0.999** | 10.416 | 0.430 | **0.03** |  |  |
| 260 | K,E(U) | 0.886 | 9.310 | 3.114 | **0.03** |  |  |
| 271 | K,E(U) | 0.992** | 10.347 | 0.922 | **0.04** |  |  |
| 282 | M,V,T(U) | 1.000** | 10.428 | 0.259 | **V(0.03)** | T(0.10) |  |
| 284 | C,R(U) | 0.501 | 5.455 | 4.980 | **0.01** |  |  |
| 289 | M,T,L(U) | 1.000** | 10.428 | 0.259 | **L(0.05)** | **T(0.02)** |  |
| 299 | M,T(U) | 1.000** | 10.428 | 0.268 | 0.07 |  |  |
| 334 | D,E(DUF) | 0.809 | 8.564 | 3.838 | **0.03** |  |  |
| 372 | L,P(Pz) | 1.000** | 10.428 | 0.259 | 0.09 |  |  |
| 375 | R,T(Pz) | 1.000** | 10.428 | 0.259 | 0.17 |  |  |
| 378 | A,V(Pz) | 1.000** | 10.428 | 0.259 | 0.59 |  |  |
| 383 | G,E,K,R(Pz) | 1.000** | 10.428 | 0.259 | E(0.06) | **K(0.02)** | R(0.07) |
| 385 | V,L(Pz) | 1.000** | 10.428 | 0.274 | 0.13 |  |  |
| 390 | T,M(Pz) | 0.873 | 9.175 | 3.283 | **0.02** |  |  |
| 533 | S,G(U) | 1.000** | 10.428 | 0.259 | 0.87 |  |  |
| 552 | S-codon(U) | 0.543 | 5.875 | 4.962 | - |  |  |
| 605 | G,A(U) | 0.998** | 10.410 | 0.496 | **0.01** |  |  |
| 700 | T,M(Pw) | 0.999** | 10.421 | 0.375 | 0.13 |  |  |
| 869 | R,K(Pw) | 1.000** | 10.428 | 0.259 | 0.07 |  |  |
| 882 | Y,C(Pw) | 0.998** | 10.413 | 0.467 | **0.03** |  |  |
| 916 | N,K(Pw) | 0.866 | 9.121 | 3.328 | 0.20 |  |  |
| 974 | Y,D(Pw) | 0.976* | 10.192 | 1.520 | **0.03** |  |  |
| Dicer 1 |  |  |  |  |  |  |  |
| 197 | Q,P(U) | 0.990** | 7.514 | 0.845 | **0.04** |  |  |
| 362 | L,V(U) | 0.961* | 7.309 | 1.428 | **0.02** |  |  |
| 384 | F,I(U) | 1.000** | 7.581 | 0.526 | **0.04** |  |  |
| 385 | P,S(U) | 1.000** | 7.582 | 0.518 | 0.83 |  |  |
| 388 | P,S(U) | 0.995** | 7.547 | 0.708 | **0.04** |  |  |
| 395 | E,K(U) | 1.000** | 7.582 | 0.518 | 0.18 |  |  |
| 497 | L,V(Hc) | 1.000** | 7.582 | 0.518 | **0.05** |  |  |
| 502 | S,L(Hc) | 0.557** | 4.432 | 3.468 | **0.02** |  |  |
| 592 | V,I(Hc) | 1.000** | 7.582 | 0.520 | 0.07 |  |  |
| 646 | K,Q(U) | 1.000** | 7.582 | 0.518 | 0.09 |  |  |
| 672 | C,Y(U) | 0.997** | 7.562 | 0.634 | 0.06 |  |  |
| 695 | E,K(U) | 0.999** | 7.577 | 0.546 | 0.08 |  |  |
| 706 | A,T(U) | 1.000** | 7.582 | 0.518 | 0.32 |  |  |
| 795 | L,M(Db) | 1.000** | 7.582 | 0.518 | 0.16 |  |  |
| 817 | M,Q,L(Db) | 1.000** | 7.582 | 0.518 | L(0.12) | Q(0.56) |  |
| 978 | I,N(Db) | 1.000** | 7.582 | 0.519 | 0.06 |  |  |
| 986 | R,K(U) | 1.000** | 7.582 | 0.518 | 0.48 |  |  |
| 1154 | S,F(U) | 1.000** | 7.582 | 0.518 | 0.11 |  |  |
| 1298 | D,E(U) | 1.000** | 7.582 | 0.518 | 0.67 |  |  |
| 1367 | D,E(U) | 1.000** | 7.582 | 0.518 | 0.42 |  |  |
| 1445 | Q,K(U) | 0.749** | 5.798 | 2.994 | **0.05** |  |  |
| 1524 | K,E(U) | 1.000** | 7.582 | 0.518 | 0.16 |  |  |
| 1535 | N,D(U) | 1.000** | 7.582 | 0.518 | 0.84 |  |  |
| 1555 | E,K(U) | 1.000** | 7.582 | 0.518 | 0.85 |  |  |
| 1575 | D,N(U) | 1.000** | 7.582 | 0.518 | 0.26 |  |  |
| 1586 | D,N(U) | 1.000** | 7.582 | 0.518 | 0.20 |  |  |
| 1616 | E,K(U) | 0.654** | 5.133 | 3.216 | 0.51 |  |  |
| 1868 | L,F(U) | 1.000** | 7.582 | 0.518 | **0.04** |  |  |
| 1901 | D,Y(U) | 1.000** | 7.582 | 0.518 | 0.11 |  |  |
| 1947 | Q,H(U) | 1.000** | 7.582 | 0.518 | 0.35 |  |  |
| 2027 | K,Q(U) | 0.892** | 6.808 | 2.163 | 0.06 |  |  |
| Dicer 2 |  |  |  |  |  |  |  |
| 25 | M,T(Dc) | 0.971* | 6.787 | 1.167 | **0.04** |  |  |
| 28 | N,K(Dc) | 1.000** | 6.975 | 0.530 | 0.09 |  |  |
| 50 | M,T(Dc) | 1.000** | 6.975 | 0.530 | 0.12 |  |  |
| 53 | D,N(Dc) | 0.998** | 6.960 | 0.609 | 0.20 |  |  |
| 75 | A,V(Dc) | 0.869** | 6.125 | 2.205 | **0.02** |  |  |
| 245 | V,I,M(U) | 1.000** | 6.975 | 0.530 | I(0.09) | **M(0.02)** |  |
| 246 | N,D(U) | 0.948** | 6.639 | 1.480 | 0.14 |  |  |
| 253 | Q,K(U) | 1.000** | 6.975 | 0.530 | 0.43 |  |  |
| 310 | Q,P(U) | 0.999** | 6.969 | 0.562 | **0.05** |  |  |
| 311 | F,I(U) | 0.998** | 6.960 | 0.606 | **0.05** |  |  |
| 399 | L,F(Dc) | 1.000** | 6.974 | 0.530 | **0.06** |  |  |
| 465 | L,F(Dc) | 0.999** | 6.970 | 0.558 | **0.04** |  |  |
| 506 | R,C(U) | 0.960* | 6.715 | 1.358 | **0.02** |  |  |
| 508 | I,V(U) | 1.000** | 6.975 | 0.530 | 0.11 |  |  |
| 524 | R,G(U) | 0.999** | 6.967 | 0.572 | **0.02** |  |  |
| 525 | R,K(U) | 0.995** | 6.943 | 0.689 | **0.04** |  |  |
| 526 | C,G(U) | 0.527** | 3.851 | 3.261 | **0.01** |  |  |
| 539 | D,H,E(U) | 1.000** | 6.974 | 0.531 | **E(0.03)** | **H(0.04)** |  |
| 541 | D,E,H(U) | 0.806** | 5.714 | 2.534 | **E(0.02)** | **H(0.01)** |  |
| 545 | E,K(U) | 0.723** | 5.172 | 2.843 | 0.06 |  |  |
| 601 | I,V(Db) | 1.000** | 6.975 | 0.530 | 0.09 |  |  |
| 743 | L,I(U) | 1.000** | 6.975 | 0.530 | 0.32 |  |  |
| 769 | E,K(U) | 1.000** | 6.975 | 0.530 | 0.15 |  |  |
| 775 | L,V(U) | 0.945** | 6.617 | 1.548 | **0.04** |  |  |
| 777 | N,D(U) | 0.995** | 6.944 | 0.679 | **0.04** |  |  |
| 779 | G,V(U) | 1.000** | 6.975 | 0.530 | 0.08 |  |  |
| 808 | C,S(U) | 0.508** | 3.727 | 3.263 | **0.02** |  |  |
| 814 | D,E(U) | 1.000** | 6.975 | 0.530 | 0.24 |  |  |
| 831 | K,E(Pz) | 1.000** | 6.975 | 0.530 | 0.29 |  |  |
| 892 | S,R,I(Pz) | 0.769** | 5.466 | 2.724 | **I(0.01)** | **R(0.02)** |  |
| 1078 | W,C(U) | 0.753** | 5.345 | 2.847 | 0.65 |  |  |
| 1123 | N,T(U) | 1.000** | 6.975 | 0.530 | 0.22 |  |  |
| 1141 | A,P(U) | 0.989* | 6.902 | 0.850 | 0.11 |  |  |
| 1223 | A,T(Rc) | 1.000** | 6.917 | 0.798 | **0.03** |  |  |
| 1446 | V,G(Hcd) | 1.000** | 6.975 | 0.530 | 0.07 |  |  |
| 1450 | L,F(Hcd) | 1.000** | 6.975 | 0.530 | 0.21 |  |  |
| 1454 | Y,D(Hcd) | 1.000** | 6.975 | 0.530 | 0.07 |  |  |
| 1492 | Y,D(Rc) | 1.000** | 6.974 | 0.531 | 0.07 |  |  |
| R3D1  26 | G,D(U) | 1.000** | 5.376 | 2.680 | 0.14 |  |  |
| 113 | K,N(U) | 0.693 | 3.532 | 2.874 | **0.01** |  |  |
| R2D2 |  |  |  |  |  |  |  |
| 38 | D,E(Db) | 0.780 | 4.562 | 2.747 | **0.02** |  |  |
| 49 | T,S(Db) | 0.993** | 5.839 | 2.057 | **0.02** |  |  |
| 83 | K,N(U) | 0.862** | 5.093 | 2.597 | 0.91 |  |  |
| 284 | L,F(U) | 0.952* | 5.643 | 2.276 | **0.05** |  |  |
| 288 | E,K(U) | 1.000** | 5.864 | 2.024 | **0.04** |  |  |
